# Autoantibodies against Protein Phosphatase Magnesium-Dependent 1A as a Biomarker for Predicting Radiographic Progression in Ankylosing Spondylitis Treated with Anti-Tumor Necrosis Factor Agents

**DOI:** 10.3390/jcm9123968

**Published:** 2020-12-07

**Authors:** Jung-Sun Lee, Eun-Ju Lee, Jae-Hyun Lee, Seok-Chan Hong, Chang-Keun Lee, Bin Yoo, Ji-Seon Oh, Sang-Hoon Lee, Tae-Jong Kim, Seung-Hun Lee, Sung-Sin Jo, Dae-Hyun Yoo, Ye-Soo Park, Tae-Hwan Kim, Yong-Gil Kim

**Affiliations:** 1 Division of Rheumatology, Department of Internal Medicine, Seoul Veterans Hospital, Seoul 05368, Korea; jungsunlee0820@gmail.com; 2 Division of Rheumatology, Department of Internal Medicine, Asan Medical Center, College of Medicine, University of Ulsan, Seoul 05505, Korea; krys72@hanmail.net (E.-J.L.); clesote@hanmail.net (J.-H.L.); medivineluke@gmail.com (S.-C.H.); cklee@amc.seoul.kr (C.-K.L.); byoo@amc.seoul.kr (B.Y.); 3 Department of Information Medicine, Asan Medical Center, Seoul 05505, Korea; doogie55@naver.com; 4 Department of Rheumatology, Kyung Hee University Hospital at Gangdong, School of Medicine, Kyung Hee University, Seoul 05278, Korea; boltaguni@gmail.com; 5 Department of Rheumatology, Chonnam National University Medical School and Hospital, Gwangju 61469, Korea; ktj1562@chonnam.ac.kr; 6 Department of Radiology, Hanyang University Hospital for Rheumatic Diseases, Seoul 04763, Korea; radsh@hanyang.ac.kr; 7 Hanyang University Institute for Rheumatology Research, Seoul 04763, Korea; joejo0517@gmail.com; 8 Department of Rheumatology, Hanyang University Hospital for Rheumatic Diseases, Seoul 04763, Korea; dhyoo@hanyang.ac.kr; 9 Department of Orthopedic Surgery, Hanyang University Hospital, Guri 11923, Korea; hyparkys@hanyang.ac.kr

**Keywords:** ankylosing spondylitis, autoantibody, biomarkers, protein phosphatase magnesium-dependent 1 A, radiographic progression

## Abstract

Background: Patients with ankylosing spondylitis (AS) have increased levels of protein phosphatase magnesium-dependent 1A (PPM1A) and autoantibodies. We evaluated the usefulness of serum anti-PPM1A antibodies as a biomarker for AS. Methods: Serum samples from 58 AS patients were obtained from a multicenter registry prior to the initiation of anti-TNF agents. The serum levels of anti-PPM1A antibodies were measured using ELISA. Spinal radiographic progression was defined as an increase in the modified stoke ankylosing spondylitis spinal score (mSASSS) by ≥2 units or a newly developed syndesmophyte. The role of exogenous PPM1A on bone mineralization was evaluated using primary osteoprogenitors acquired from patients with AS and non-inflammatory controls. Results: The baseline levels of anti-PPM1A antibodies and mSASSS were higher in the radiographic progression group than in the non-progression group. In logistic regression analysis, baseline mSASSS and serum anti-PPM1A antibodies were associated with a higher risk of progression. The level of anti-PPM1A antibodies for predicting progression had an AUC of 0.716 (cut-off value: 43.77 ng/mL). PPM1A stimulation increased matrix mineralization in AS-osteoprogenitors but not in controls. Conclusion: Along with mSASSS, the serum levels of anti-PPM1A antibodies might be useful as a predictor of radiographic progression after treatment with anti-TNF agents.

## 1. Introduction

Ankylosing spondylitis (AS) is chronic inflammatory arthritis characterized by ankylosis of the spine and sacroiliac joint [1]. Spinal ankylosis in AS causes functional disability, resulting in a socioeconomic loss at a young age [2]. Recently, tumor necrosis factor (TNF) inhibitors have been considered as an effective treatment for AS; however, despite their wide use, it remains uncertain whether TNF inhibitors can prevent spinal progression [3,4].

Several serologic biomarkers have been shown to have clinical utility in AS, including HLA-B27 for diagnosis and erythrocyte sedimentation rate (ESR) and C-reactive protein (CRP) for disease activity [5,6]. However, there is currently no available biomarker to detect structural damage caused in AS. Since AS structural damage is irreversible, biomarker detection at the early stages of the disease will be important to identify patients who are at the most risk of developing structural damage.

Protein phosphatase magnesium-dependent 1 A (PPM1A), a serine/threonine protein phosphatase, regulates bone morphogenetic protein (BMP) and Wingless (Wnt) signaling pathway, and overexpression of PPM1A enhances osteoblast differentiation [7,8,9]. Our previous study reported that the serum levels of anti-PPM1A antibodies are higher in AS than in other autoimmune diseases [10]. Furthermore, the serum levels of anti-PPM1A antibodies were higher in AS patients with high-grade sacroiliitis than those with low-grade sacroiliitis. Interestingly, PPM1A was expressed in AS synovial tissue, and intracellular PPM1A overexpression promoted osteoblast differentiation. However, anti-PPM1A antibodies have not yet been implicated clinically because the levels were not measured quantitatively.

Here, we developed a method for quantitative measurement of the serum anti-PPM1A antibody levels and evaluated the usefulness of serum anti-PPM1A antibodies as a biomarker of spinal progression in AS patients beginning treatment with anti-TNF agents.

## 2. Experimental Section

### 2.1. Study Population

This multicenter cohort study included AS patients who took part in a prospective observational study (ClinicalTrials.gov Identifier: NCT02557308) for the evaluation of the safety and effectiveness of anti-TNF agents, including infliximab, etanercept, golimumab, and adalimumab between October 2014 and September 2015 in South Korea. We included patients who agreed to provide their clinical information and blood samples for a similar research scope and comprehensive research purpose. All included patients met the 1984 modified New York classification criteria [11] and were anti-TNF agent-naïve. Patients with no measurable images of the cervical spine and lumbar spine at the start of anti-TNF treatment and follow-up were excluded. Serum samples and clinical information were collected before treatment with anti-TNF agents. Collected clinical information included the following: age; sex; Bath Ankylosing Spondylitis Disease Activity Index (BASDAI); Bath Ankylosing Spondylitis Functional Index (BASFI) score; Ankylosing Spondylitis Disease Activity Score (ASDAS); radiographic finding; and laboratory findings, including ESR, and CRP.

### 2.2. Measurement of Anti-PPM1A Antibodies Levels

The levels of anti-PPM1A antibodies in sera were quantified as follows. Nunc-Immuno-Maxisorp 96-well plates were coated with 1 μg/mL of PPM1A (YbdY, Seoul, Korea) in PBS at 4 °C overnight. The plates were washed with PBS-Tween 20 (PBST; 0.05%, *v*/*v*), and then blocked with 2% Chon Block (Chondrex, Inc., Redmond, WA, USA) in 0.05% PBST for 1 h at room temperature. After washing, 100 μL of serum diluted with 2% Chon Block (1:50) in 0.05% PBST was added and incubated for 1 h at room temperature. For standards, 10 μg/mL of anti-PPM1A (Abnova, Taipei, Taiwan) was serially diluted 2-fold in 2% Chon Block in 0.05% PBST. After washing, HRP-conjugated protein G (Thermo Fisher Scientific, Waltham, MA, USA) was added and incubated for 1 h at room temperature. The reactions were developed with the 3,3′,5,5′-tetramethylbenzidine (TMB) substrate for 15 min at room temperature. The color reactions were stopped with 50 μL H2SO4 and the optical density was read at 450 nm.

### 2.3. Evaluation of Radiographic Progression

The radiographs of the lateral cervical spine and lumbar spine were taken at two time points (baseline and follow-up) and were reviewed and graded by an independent radiologist. After blinding the radiographs for the patient’s identity and the time point, all images of the cervical spine and lumbar spine were scored using the modified stoke ankylosing spondylitis spinal score (mSASSS). Spinal radiographic progression was defined as an increase in the mSASSS by ≥2 units or a newly developed syndesmophyte, defined as mSASSS of at least 2 at a vertebral level, with a score of 0 or 1 at baseline [12] 

### 2.4. Primary Human Osteoprogenitors

Studies involving human materials were performed in compliance with the Helsinki Declaration and approved by the Ethics Committee of Hanyang University Hospital; written informed consent was obtained from all subjects (IRB-2014-05-002). Human bone tissues were obtained during spine surgery from the facet joints of 3 patients with AS and 4 patients with non-inflammatory spinal conditions such as car accident injuries and spinal compression diseases as control. Bone-derived osteoprogenitors were obtained from facet joints of patients. Isolation and characterization of osteoprogenitors were performed as described previously [13,14]. Briefly, osteoprogenitors were seeded in growth medium and then stimulated with osteogenic medium (OM) containing ascorbic acid, beta-glycerol phosphate, and dexamethasone. The OM was changed every three days. Alkaline phosphatase (ALP) was assessed using ALP activity colorimetric assay kit (K412, Biovision, San Francisco, CA, USA) and ALP staining (85L2, Sigma, St. Louis, MO, USA). For the assessment of the matrix mineralization, matrix mineralization was visualized using several staining methods including Alizarin red staining (ARS; A5533, Sigma, St. Louis, MO, USA) for calcium deposition, and hydroxyapatite staining (HA; PA-1503, Lonza, Basel, Basel-stadt, Switzerland) for hydroxyapatite formation. For ARS quantification, the stained wells were extracted with absolute acetic acid at 37 °C. for 30 min followed by centrifugation. The supernatant was transferred to a white 96 wells plate and read at the excitation wavelength of 405 nm with ELISA plate reader. For HA quantification, the stained cells were read at an excitation wavelength of 492 nm and an emission wavelength of 550 nm with an ELISA plate reader.

### 2.5. Statistical Analysis

Continuous values are expressed as mean (standard deviations) for parametric data, or as median (interquartile range) for nonparametric data. Mann-Whitney U-test was used to compare the clinical parameters in the radiographic progression group and non-progression group. Logistic regression analysis was performed to identify the factors associated with spinal radiographic progression, and the odds ratios (ORs) and 95% confidence intervals (95% CIs) were reported. Receiver operating characteristics (ROC) analysis was used to determine the predictive value of factors that were associated with spinal radiographic progression. The cut-off value was determined at the level where Youden’s index was maximum [15]. A *p*-value of < 0.05 was considered statistically significant in all analyses. All analyses were conducted using IBM SPSS Version 20.0 (IBM Corp., Armonk, NY, USA).

## 3. Results

### 3.1. Characteristics of Patients

Among the 153 patients in the prospective observational study, 58 patients who had measurable baseline and follow-up images of the cervical spine and lumbar spine were included in this study. As shown in Table 1, more than 80% of patients were male, and the mean age of the patients was 37.8 ± 10.9 years. The median disease duration at the initiation of anti-TNF agent treatment was 14 (4.0–85.8) months, and the median baseline mSASSS was 10 (6.8–24) units. Anti-TNF agents were used in the order of infliximab (46.6%), etanercept (24.1%), golimumab (22.4%), and adalimumab (6.9%).

### 3.2. Radiographic Progression and Clinical Parameters

**Figure 1:**
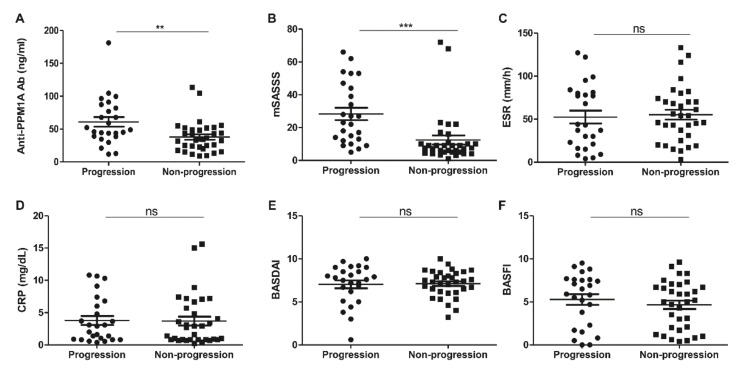
(**A**) Anti-PPM1A antibodies, (**B**) mSASSS, (**C**) ESR, (**D**) CRP, (**E**) BASDAI, and (**F**) BASFI at baseline in patients with radiographic progression consequently. PPM1A: Protein phosphatase magnesium-dependent 1A, mSASSS: Modified Stoke Ankylosing Spondylitis Spinal Score, ESR: Erythrocyte Sedimentation Rate, CRP: C-reactive protein, BASDAI: Bath Ankylosing Spondylitis Disease Activity Index, BASFI: Bath Ankylosing Spondylitis Functional Index. ** *p* < 0.01, *** *p* < 0.001, ns: Not significant.

The median follow-up mSASSS was 11.5 (7–28.5) units, and the median time to follow-up mSASSS was 22 (20–25) months. A total of 58 patients maintained anti-TNF agent treatment, among whom, 43.1% (25/58) showed spinal radiographic progression (Table 1). The patients with radiographic progression showed higher baseline anti-PPM1A antibody levels and baseline mSASSS than patients without radiographic progression (Table 1 and Figure 1A,B). However, the serum level of anti-PPM1A antibodies was not correlated with baseline mSASSS (r = −0.001, *p* = 0.993). Other baseline parameters, including ESR, CRP, BASDAI, and BASFI, were not different between the radiographic progression group and the non-progression group (Figure 1C–F). In the subgroup analysis among the patients having grade 2 sacroiliitis, baseline mSASSS was higher in the progression group than the non-progression group (*p* = 0.03), but other baseline parameters were not different.

### 3.3. Factors Associated with Spinal Radiographic Progression

Logistic regression analysis was performed to evaluate the clinical factors associated with radiographic progression (Table 2). Univariate analysis indicated that male, age, baseline mSASSS, and baseline serum level of anti-PPM1A antibodies were associated with a higher risk of radiographic progression; however, being a current smoker was not associated with radiographic progression. In multivariable analysis, baseline mSASSS (OR, 1.083; 95% CI, 1.013–1.159; *p* = 0.019) and serum level of anti-PPM1A antibodies (OR, 1.045; 95% CI, 1.011–1.080; *p* = 0.010) were associated with spinal radiographic progression.

### 3.4. Predictive Value of Serum Anti-PPM1A Antibodies and Baseline mSASSS for Spinal Radiographic Progression

**Figure 2:**
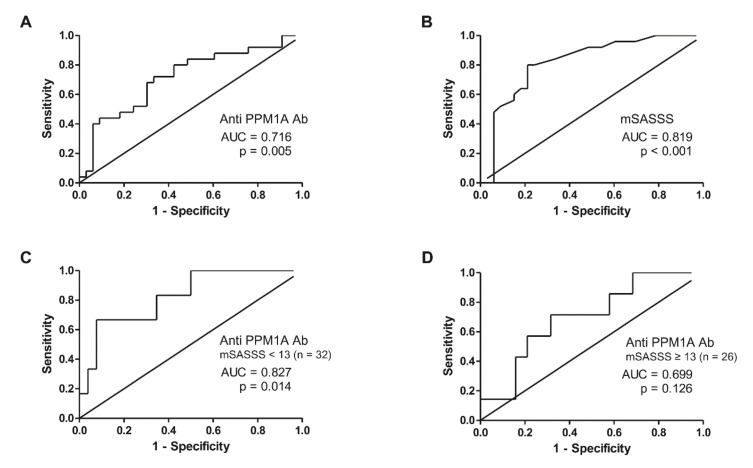
ROC analysis predicting radiographic progression in AS patients treated with anti-TNF agents with regards to (**A**) anti-PPM1A antibodies, (**B**) mSASSS, (**C**) anti-PPM1A antibodies in patients with mSASSS < 13 units, and (**D**) anti-PPM1A antibodies in patients with mSASSS ≥ 13 units. ROC: Receiver operating characteristic, TNF: Tumor necrosis factor, PPM1A: Protein phosphatase magnesium-dependent 1A, AUC: Area under the curve, mSASSS: Modified Stoke Ankylosing Spondylitis Spinal Score.

The ROC analysis result is shown in Figure 2. The serum level of anti-PPM1A antibodies had an area under the curve (AUC) of 0.716 (95% CI, 0.580–0.852) at a cut-off value of 43.77 ng/mL (sensitivity 68%, specificity 70%). It showed higher accuracy for predicting radiographic progression when analyzed in males (Supplementary Figure S1). The baseline mSASSS had an AUC of 0.819 (95% CI, 0.708–0.931) at a cut-off value of 13 units (sensitivity 76%, specificity 78%). When subgroup analysis was performed according to the cut-off of the mSASSS, the predictive accuracy of anti-PPM1A antibodies was increased in patients with an mSASSS less than 13 (AUC, 0.827; 95% CI, 0.653–1.000) (Figure 2C).

**Figure 3:**
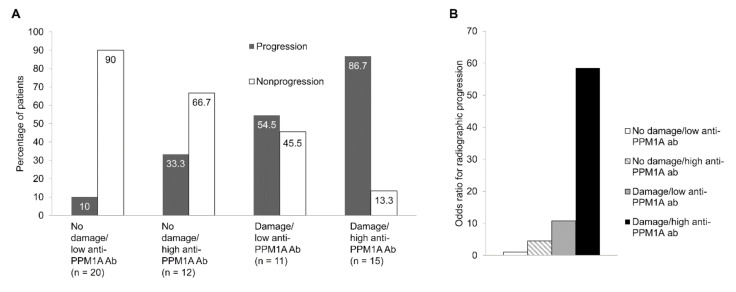
(**A**) Frequency of radiographic progression, (**B**) odds ratio for radiographic progression categorized according to anti-PPM1A antibodies and mSASSS at baseline. Damage and high anti-PPM1A antibodies were defined as mSASSS ≥13 units and anti-PPM1A antibodies ≥43.77 ng/mL, respectively. PPM1A: Protein phosphatase magnesium-dependent 1A, mSASSS: Modified Stoke Ankylosing Spondylitis Spinal Score.

When the patients were divided into four groups based on the cut-off of serum anti-PPM1A antibodies and the baseline mSASSS, patients with a high level of serum anti-PPM1A antibodies, defined as ≥43.77 ng/mL, and damage, defined as mSASSS ≥13 units at baseline, showed the highest frequency of radiographic progression (Figure 3A). Logistic regression analysis also showed that high anti-PPM1A antibodies and damage at baseline were associated with a higher risk of radiographic progression compared to low anti-PPM1A antibodies and no damage at baseline (OR, 58.5; *p* < 0.001) (Figure 3B).

### 3.5. PPM1A Induces Matrix Mineralization of AS-Osteoprogenitor

**Figure 4:**
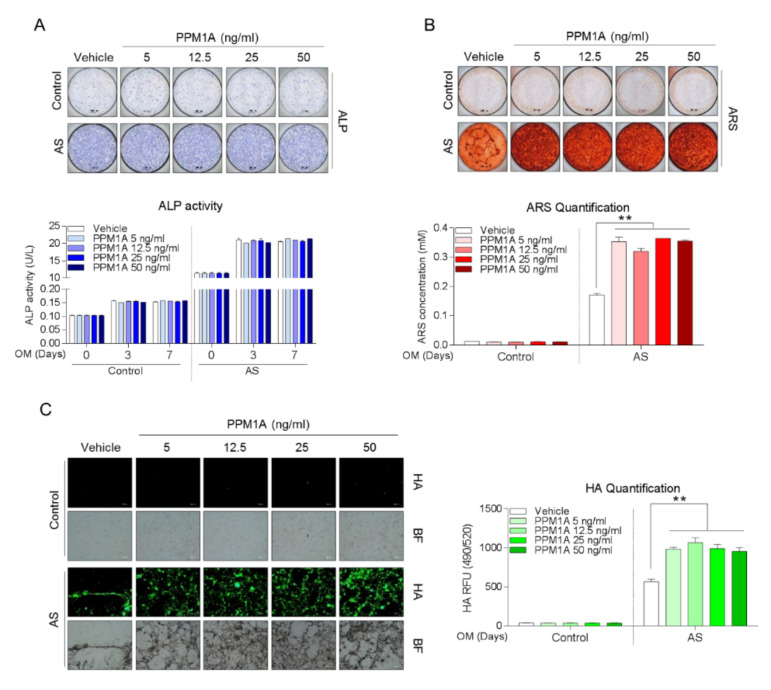
Both control- (*n* = 4) and AS-osteoprogenitors (*n* = 3) were stimulated with as indicated PPM1A dose during osteogenic differentiation, and subjected to analysis for (**A**) ALP staining and activity at 0, 3, and 7 days, (**B**) ARS staining and quantification at 21 days, and (**C**) Hydroxyapatite staining and quantification at 21 days. Data are presented as means + SD; ** *p* < 0.01 by unpaired two-tailed *t*-tests. AS: Ankylosing spondylitis, PPM1A: Protein phosphatase magnesium-dependent 1A, ALP: Alkaline phosphatase, ARS: Alizarin Red S, HA: Hydroxyapatite.

There was no effect of exogenous PPM1A stimulation on ALP activity of both control- and AS-osteoprogenitors (Figure 4A). However, as shown in Figure 4B,C, AS-osteoprogenitors showed matrix mineralization features and its quantification was significantly increased by PPM1A stimulation, whereas those in control-osteoprogenitors were not changed. These data suggested that extracellular PPM1A might be responsible for matrix mineralization in AS patients particularly, although the biologic role of anti-PPM1A antibodies was not defined.

## 4. Discussion

The present study demonstrates that the serum level of anti-PPM1A antibodies is associated with a higher risk of spinal radiographic progression in patients at the initial stage of treatment with anti-TNF agents, along with baseline mSASSS.

Several studies have determined clinical parameters that are useful for the prediction of radiographic progression in AS patients [16,17,18]. That is, the presence of syndesmophytes at baseline, increased acute phase reactants, male sex, older age, presence of uveitis, and smoking are considered to be predictors of radiographic progression. Furthermore, a 12-year long-term study demonstrated that radiographic progression occurred more severely in HLA-B27 positive male patients, and faster in patients with an mSASSS ≥10 at baseline [19]. The present study showed that a high baseline mSASSS was strongly associated with radiographic progression during anti-TNF agent treatment, which is a similar result to those of the aforementioned studies. Moreover, we demonstrated that high serum levels of anti-PPM1A antibodies were associated with a high risk of progression. These findings suggest that anti-PPM1A antibodies could be an independent biomarker of radiographic progression in AS with baseline mSASSS.

Several studies have reported the association between serum biomarkers and radiographic progression in AS patients. Indeed, serum matrix metalloproteinase 3, a cartilage turnover marker, has been proposed as a predictor of disease progression in AS, especially in patients with pre-existing radiographic damage [20]. In other studies, the serum levels of sclerostin or dickkopf-1, inhibitory molecules of Wnt signaling, and serum calprotectin could predict the formation of syndesmophytes in AS patients [13,21,22]. Previously, endogenous PPM1A could be induced by TNF-α [23], and the endogenous overexpression of PPM1A in preosteoblasts (MC3T3-E1) was shown to enhance the differentiation of osteoblasts independently of the Wnt/β-catenin or BMPs pathway [10]. Interestingly, in the present study, the bone mineralization was promoted by exogenous PPM1A in AS-osteoprogenitors, but not in non-inflammatory controls. Although the role of anti-PPM1A antibodies on bone pathology was not defined directly, the association between the radiographic progression and serum level of anti-PPM1A antibodies may be explained.

There is still controversy surrounding whether anti-TNF agents can prevent radiographic progression in AS [3,4]. Previous clinical trials showed no beneficial effect of anti-TNF agents on radiographic progression [24,25,26]; however, some studies have reported that radiographic progression in AS could be delayed, especially following long-term use of anti-TNF agents [13,27]. Furthermore, use of anti-TNF agents in the early stages of AS could decrease the radiographic progression by effective suppression of inflammation [28]. We considered that the measurement of anti-PPM1A antibodies and the mSASSS could be applied in practice before initiating anti-TNF treatment. In patients without damage, but with a high level of anti-PPM1A antibodies at baseline, a physician might expect the development of radiographic progression in one third of patients during treatment with anti-TNF agents. In other situations with a high level of anti-PPM1A antibodies and damage at baseline, treatment with non-TNF agents may be considered since this group shows the highest risk of radiographic progression during use of anti-TNF agents.

Our study has several limitations. First, the number of patients was small to reach a powerful conclusion, and only patients who were treated with anti-TNF agents were enrolled. Moreover, more than 40% of patients showed progression during the study period that might result in some selection bias toward patients at higher risk of progression. Further studies with a larger number of patients with varying degrees of disease activity are needed to confirm the present findings. Second, the follow-up radiographs of the lateral cervical spine and lumbar spine were performed at 22 (20–25) months, which was a relatively short time to evaluate spinal radiographic progression. Finally, mSASSS was evaluated by one radiologist thus, we could not provide the inter-observer reliability.

## 5. Conclusions

Anti-PPM1A antibody levels are correlated with disease activity and may be useful as a biomarker for predicting radiographic progression in AS patients before initiating treatment with anti-TNF agents.

## Figures and Tables

**Table 1:** Baseline characteristics of patients before treatment with anti-TNF agents.

	All Patients (*n* = 58)	Progression (*n* = 25)	Non-Progression (*n* = 33)	*p*
Male, *n* (%)	47 (81)	24 (96.0)	23 (69.7)	0.016
Age (years)	37.8 ± 10.9	41.5 ± 11.2	35 ± 9.8	0.021
Smoking status				0.841
Current smoker	19 (32.8)	9 (36)	10 (30.3)	
Never smoker	25 (43.1)	11 (44)	14 (42.4)	
Ex-smoker	14 (24.1)	5 (20)	9 (27.3)	
Disease duration (months)	14 (4.0–85.8)	29 (3.5–124)	9 (5–67.5)	0.588
Sacroiliitis				0.175
Grade 2	22 (37.9)	7 (28)	15 (45.5)	
Grade 3 or 4	36 (62.1)	18 (72)	18 (54.5)	
ESR (mm/h)	54.0 ± 34.4	52.4 ± 37.5	55.2 ± 32.4	0.759
CRP (mg/dL)	2.8 (0.8–6.2)	2.9 (0.9–6.4)	2.7 (0.8–6.2)	0.654
BASDAI	7.5 (6.2–8.5)	7.6 (5.7–8.8)	7.3 (6.2–8.4)	0.649
BASFI	5.7 (1.9–7.3)	5.9 (2.0–7.6)	5.0 (1.9–6.9)	0.350
ASDAS-CRP	3.2 ± 0.9	3.1 ± 0.9	3.2 ± 0.9	0.723
mSASSS	10 (6.8–24)	23 (12.5–45.5)	8 (5–10.5)	<0.001
Anti-PPM1A Abs (ng/mL)	43.5 (24.9–56.3)	48.9 (38.9–84.6)	34.3 (21.2–50.2)	0.005
NSAIDs use				0.100
None	4 (6.9)	3 (12)	1 (3)	
On demand	22 (37.9)	6 (24)	16 (48.5)	
Regular use	32 (55.2)	16 (64)	16 (48.5)	
Anti-TNF agents				
Infliximab	27 (46.6)	9 (36)	18 (54.5)	0.161
Etanercept	14 (24.1)	9 (36)	5 (15.2)	0.066
Golimumab	13 (22.4)	5 (20.0)	8 (24.2)	0.701
Adalimumab	4 (6.9)	2 (8)	2 (6.1)	1.000

Values are presented as mean ± standard deviation or median (interquartile range). AS: ankylosing spondylitis, TNF: tumor necrosis factor, ESR: Erythrocyte sedimentation rate, CRP: C-reactive protein, BASDAI: Bath Ankylosing Spondylitis Disease Activity Index, BASFI: Bath Ankylosing Spondylitis Functional Index, ASDAS: ankylosing Spondylitis Disease Activity Score, mSASSS: Modified Stoke Ankylosing Spondylitis Spinal Score, PPM1A: Protein phosphatase magnesium-dependent 1A, NSAIDs: Non-steroidal anti-inflammatory drugs.

**Table 2:** Factors associated with radiographic progression in AS patients treated with anti-TNF agents.

	Univariate			Multivariable		
	OR	CI	*p*	OR	CI	*p*
Male	10.435	1.235–88.133	0.031	11.760	0.848–163.066	0.066
Age	1.062	1.007–1.120	0.026			
Current Smoker	1.294	0.429–3.901	0.647			
Disease Duration	1.004	0.998–1.011	0.173			
ESR	0.998	0.982–1.013	0.754			
CRP	1.007	0.875–1.159	0.922			
High-grade sacroiliitis	2.143	0.706–6.501	0.178			
BASDAI	0.979	0.741–1.293	0.881			
BASFI	1.079	0.899–1.295	0.417			
mSASSS	1.057	1.017–1.099	0.005	1.083	1.013–1.159	0.019
Anti-PPM1A Abs	1.029	1.006–1.053	0.012	1.045	1.011–1.080	0.010
Length of treatment with anti-TNF agents	0.986	0.791–1.015	0.085			
NSAIDs regular use	1.889	0.652–5.476	0.242			
Infliximab	0.469	0.161–1.361	0.164			
Etanercept	3.150	0.899–11.038	0.073			
Golimumab	0.781	0.221–2.761	0.702			
Adalimumab	1.348	0.177–10.292	0.774			

AS: ankylosing spondylitis, TNF: tumor necrosis factor, OR: Odds ratio, CI: Confidence interval, ESR: Erythrocyte sedimentation rate, CRP: C-reactive protein, BASDAI: Bath Ankylosing Spondylitis Disease Activity Index, BASFI: Bath Ankylosing Spondylitis Functional Index, mSASSS: Modified Stoke Ankylosing Spondylitis Spinal Score, PPM1A: Protein phosphatase magnesium-dependent 1A, NSAIDs: Non-steroidal anti-inflammatory drugs.

## Supplementary Materials

The following are available online at https://www.mdpi.com/2077-0383/9/12/3968/s1, Figure S1: ROC analysis according to sex predicting radiographic progression in AS patients treated with anti-TNF agents with regards to anti-PPM1A antibodies.
