# Supplementary figures and images for: Autoantibodies against Protein Phosphatase Magnesium-Dependent 1A as a Biomarker for Predicting Radiographic Progression in Ankylosing Spondylitis Treated with Anti-Tumor Necrosis Factor Agents
Source: J Clin Med. 2020 Dec 7;9(12):3968. doi: 10.3390/jcm9123968 (PMC7762424; doi:10.3390/jcm9123968)

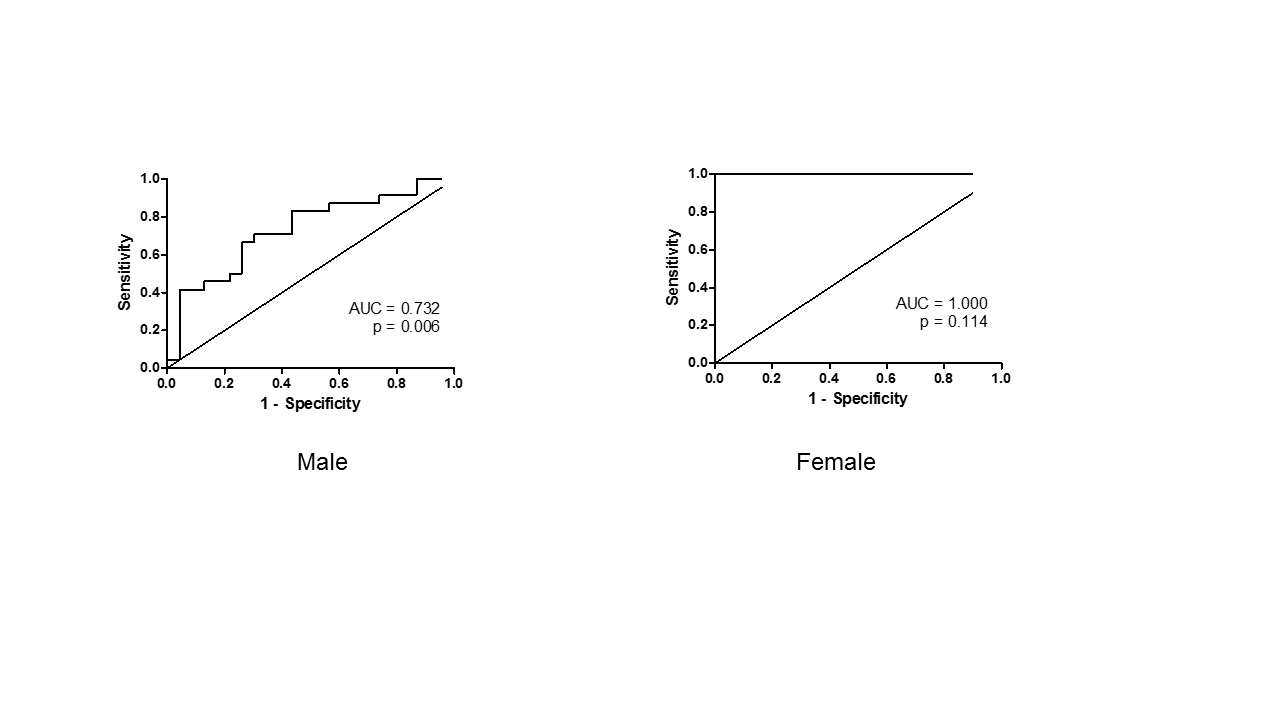

Supplement: Supplementary file 1 [file jcm-09-03968-s001.tif]
